# Quaternion-Based Local Frame Alignment between an Inertial Measurement Unit and a Motion Capture System

**DOI:** 10.3390/s18114003

**Published:** 2018-11-16

**Authors:** Jung Keun Lee, Woo Chang Jung

**Affiliations:** Inertial Motion Capture Lab, Department of Mechanical Engineering, Hankyong National University, Anseong 17579, Korea; chwch93@hknu.ac.kr

**Keywords:** local frame alignment, quaternion, inertial measurement unit, motion capture system, angular velocity

## Abstract

Local frame alignment between an inertial measurement unit (IMU) system and an optical motion capture system (MCS) is necessary to combine the two systems for motion analysis and to validate the accuracy of IMU-based motion data by using references obtained through the MCS. In this study, we propose a new quaternion-based local frame alignment method where equations of angular velocity transformation are used to determine the frame alignment orientation in the form of quaternion. The performance of the proposed method was compared with those of three other methods by using data with different angular velocities, noises, and alignment orientations. Furthermore, the effects of the following three factors on the estimation performance were investigated for the first time: (i) transformation concept, i.e., angular velocity transformation vs. angle transformation; (ii) orientation representations, i.e., quaternion vs. direction cosine matrix (DCM); and (iii) applied solvers, i.e., nonlinear least squares method vs. least squares method through pseudoinverse. Within our limited test data, we obtained the following results: (i) the methods using angular velocity transformation were better than the method using angle transformation; (ii) the quaternion is more suitable than the DCM; and (iii) the applied solvers were not critical in general. The proposed method performed the best among the four methods. We surmise that the fewer number of components and constraints of the quaternion in the proposed method compared to the number of components and constraints of the DCM-based methods may result in better accuracy. Owing to the high accuracy and easy setup, the proposed method can be effectively used for local frame alignment between an IMU and a motion capture system.

## 1. Introduction

Recent advances in micro-electro-mechanical systems (MEMS) technology, as well as mobile and ubiquitous computing, have fostered considerable amount of interest in the inertial measurement unit (IMU) as a wearable motion sensor [1,2,3]. A typical IMU consists of a tri-axial accelerometer and a tri-axial gyroscope that measure the linear acceleration and angular velocity of the unit, respectively, with respect to the local frame fixed to the unit. Since the wearable IMUs can overcome limited working volume issue and occlusion problem of typical camera-based measurements, they have tremendous benefits for various applications such as the practice of physical medicine and rehabilitation [4,5,6] and motion reconstruction for computer animation productions [7,8,9,10,11,12,13].

Even if IMUs have great potential as motion tracking sensors, position and orientation information, which are required in motion analysis, are not directly provided by IMUs. Instead, the information has to be estimated through sophisticated sensor fusion algorithms [14,15,16]. Accordingly, with regard to IMU-based applications, evaluation of the estimated quantities is performed by comparing them to references obtained through a high framerate marker-based optical motion capture system (MCS), like VICON, which is often considered as a gold standard in human motion analysis [17,18]. This is because the marker-based MCS directly measures the positions of markers and can thus provide position and orientation with very high accuracy [4,16].

During evaluation, alignment between the local frame of the IMU and the local frame of the MCS is necessary. Furthermore, when an IMU is combined with an MCS for any reason, the local frames of the two systems must be aligned to compare IMU data with MCS data. In general, manufacturers align the local IMU frame to the IMU casing (or housing). Therefore, in most cases, users manually align the MCS local frame to the casing with careful attention. However, this process is prone to alignment errors. In addition, the manufacturer’s alignment of the IMU frame to the casing has an error of a few degrees (e.g., <3° in [19]).

The purpose of this article is twofold: (i) to propose a quaternion-based local frame alignment method between an IMU and an MCS, and (ii) to investigate the effects of the angular velocity, noise, and alignment orientation to be estimated on the estimation performance. Regarding the first purpose, the novelty of the proposed method is that while the method in [20] uses a DCM with nine components to be determined, the proposed method uses a quaternion with only four components. Regarding the second purpose, the aforementioned effects have not been investigated in the literature.

## 2. Related Works

Recently, this issue was discussed in a few research studies [20,21,22]. De Vries et al. [20] introduced a method for the local frame alignment of an IMU with an MCS in the appendix of [20]. This method is based on the fact that angular velocities expressed in the IMU frame are transformed to those expressed in the MCS frame by pre-multiplying the constant alignment rotation matrix or direction cosine matrix (DCM) of the IMU frame with respect to the MCS frame. However, since the research in [20] focused on the evaluation of the effect of magnetic distortion on IMU applications, the performance of the alignment method was not evaluated.

Chardonnens et al. [21] proposed another alignment method using the gyroscopic angles of the two frames by time-integrating the angular velocity measurements. Then, the alignment DCM between the IMU and MCS frames was determined by a frame transformation equation as in [20]. A nonlinear least squares Levenberg–Marquardt algorithm [23] was used to solve the problem. Neither of the above two methods requires any alignment device or information about the IMU casing. This makes the methods easy to perform and practical.

Mecheri et al. [22] evaluated eight methods for the orientation alignment of two coordinate systems. The eight methods include the methods in [20] and [21] as well as methods under the theme “hand-eye calibration” or the “AX = XB” problem in robotics [24,25,26,27,28,29]. In fact, in order to apply the hand-eye calibration methods to the frame alignment between an IMU and an MCS (which is of interest here), very accurate orientation of the IMU with respect to its inertial reference frame should be available, which is not the case in most IMU applications. Mecheri et al. recommended the method in [20] rather than the method in [21] as the former had better performance in an evaluation.

In [21,22], there are a couple of interesting comments regarding the comparison of the two methods [20,21]. First, regarding the comment on the signal-to-noise (S2N) ratio (which is referred to as *C1*, henceforth), [21] argued that the gyroscopic angle provides a better S2N ratio than the angular velocity or acceleration and does not require fine filtering, which might be sensitive to the execution of the alignment movement. However, [22] disagreed with the argument as the method in [20] performed better than the method in [21]. Second, regarding the comment on the false local minima of the non-linear least squares method (which is referred to as *C2*), [22] stated that, while the method in [20] uses a linear least squares method through pseudoinverse matrix operation to calculate the alignment DCM, the method in [21] uses a non-linear least squares method that could partially contribute to the performance discrepancies between the two methods. However, [22] did not verify for the above two comments. *C1* can be investigated by inserting a predefined level of noise to angular velocity measurements and generating different S2N ratio data. *C2* can be verified by evaluating the estimation performance for several different alignment orientations to be estimated, while the initial orientation for the non-linear least squares method is fixed (i.e., it is not varied).

## 3. Method

### 3.1. Alignment Procedure

For the frame alignment of an IMU coordinate system {*I*} to an MCS coordinate system {*M*}, the proposed method uses a quaternion q, which is defined as the sum of a scalar $q_{0}$ and a vector **e** = ${\left[q_{1}\;q_{2}\;q_{3}\right]}^{T}$, i.e., q = ${\left[q_{0},\;e^{T}\right]}^{T}$. Any three-dimensional column vector a can be represented as the quaternion $\hat{a}$ with the scalar part set to zero, i.e., $\hat{a}={\left[0,\;a^{T}\right]}^{T}$.

Then, the angular velocities expressed in the IMU frame are transformed to those expressed in the MCS frame by the following equation that includes the constant alignment quaternion q:

(1) $${}^{M}\hat{\omega }{}_{i}=q\;{}^{I}\hat{\omega }{}_{i}\;q^{*},\;i=1\ldots \ldots N$$

where *i* indicates the frame number; *N* is the number of frames; and $q^{*}$ is the conjugate of q, defined as $q^{*}$ = ${\left[q_{0},\;-e^{T}\right]}^{T}$. In addition, ${}^{I}\hat{\omega }{}_{i}$ is the quaternion form of the angular velocity measured with an IMU (i.e., gyroscope signals), ωIi=[ωIx,i, ωIy,i, ωIz,i]T, and ${}^{M}\hat{\omega }{}_{i}$ is the quaternion form of the angular velocity as derived from MCS data, ωMi=[ωMx,i, ωMy,i, ωMz,i]T. Note that three markers of the MCS form a plane and can define a unique local frame. Accordingly, the MCS can track the quaternion of the MCS local frame with respect to the MCS global frame qM. Then, ${}^{M}\hat{\omega }{}_{i}$ is obtained as:

(2) Mω^i=2 qMi* q.Mi

where q.M is the time derivative of qM obtained by the numerical differentiation and may cause derivative noises in ${}^{M}\hat{\omega }{}_{i}$. Equation (1) can be rewritten as:

(3) $${}^{M}\hat{\omega }{}_{i}\;q=q\;{}^{I}\hat{\omega }{}_{i},\;i=1\ldots \ldots N$$

By combining Equation (3) with the quaternion normalization constraint ||q||=1, the following four non-linear constraint functions, $F_{i}$, are derived:

(4) Fi=[(ωMx,i−ωIx,i)q0−(ωMz,i+ωIz,i)q2+(ωMy,i+ωIy,i)q3(ωMy,i−ωIy,i)q0+(ωMz,i+ωIz,i)q1−(ωMx,i+ωIx,i)q3(ωMz,i−ωIz,i)q0−(ωMy,i+ωIy,i)q1+(ωMx,i+ωIx,i)q2w×(q02+q12+q22+q32−1)]=0,    i=1……N

where w is the weighting factor for the normalization constraint which was set to 10 in this paper.

In this study, MATLAB function *fsolve* is used to solve the non-linear Equation (4), with the fixed initial quaternion ${\left[1,\;0,\;0,\;0\right]}^{T}$ and the specified algorithm option, ”*Levenberg–Marquardt*,” which is chosen in [21]. The solution from the *fsolve* is finally normalized to be meaningful as an orientation representation. Source codes and example data are available as Supplementary Materials.

### 3.2. Validation Using Simulated Data

A triaxial gyroscope in a wireless MTw IMU system (Xsens Technologies, Enschede, The Netherlands) was used to obtain the angular velocities with respect to the IMU frame ωI at a 100-Hz sampling rate. Before measurement, the IMU was stabilized following a few minutes of sensor warm-up time, which makes effect of ambient temperature on measurement minimal. The gyroscope in an MTw system provides the so-called ‘calibrated sensor readings’ for orthogonality in a body-fixed coordinate system which is defined as the IMU frame. The gyroscope has a full scale of ±2000°/s and a noise of 0.01°/s/√Hz. See [30] for detailed specification of the MTw. Within a volume of 30 cm radius, the MTw was moved by hand in a random manner in three-dimensional space as evenly as possible, as in [20] and [22]. The alignment movement had better cover the rotation space as evenly as possible because the solvers such as least square methods minimize the total residual, as discussed in [21]. The Awinda USB dongle station connected to a PC received wireless data from the IMU.

In actual alignment processes, angular velocities with respect to the MCS frame ωM are derived from marker data based on Equation (2). However, in the validation of this chapter, ωM’s were generated (or simulated) by using the above ωI’s and a predefined q which was to be estimated, according to Equation (1). Therefore, only the MTw sensor without an MCS was used for this validation. This is because, if the validation test is performed using an MCS as in the actual process, it is impossible to acquire the perfectly exact alignment orientation q. Note that the exact alignment orientation as a reference should be available for accurate calculation of estimation errors. Furthermore, the position tracking errors of an MCS may interfere with the performance comparison between alignment methods. For these reasons, the simulated angular velocity data according to the reference alignment orientation that we set were considered as more suitable for the performance comparison between methods.

Five different tests were conducted depending on the averaged amount of angular velocity: T1 (1.5<||ω||<2.5 rad/s, very slow), T2 (3.5<||ω||<4.5 rad/s, slow), T3 (5.5<||ω||<6.5 rad/s, medium), T4 (7.5<||ω||<8.5 rad/s, fast), and T5 (9.5<||ω||<10.5 rad/s, very fast). This was to observe the effect of amount of angular velocity on the alignment estimation performance.

Ten trials were performed for each test, and each trial lasted over 60 s as in [22]. A cutoff threshold of 0.2 rad/s was applied as in [20] since angular velocities should be above the noise level of the gyroscope. Then, each trial was set to 6000 samples of data after cutoff, to provide a sufficient amount of data for solvers. Note that sampling rate is not critical for the proposed method, as long as a sufficient amount of data can be provided to the method.

In order to observe the effect of the signal noisiness on the estimation performance, four levels of random noises were added to both ωI and ωM: N1 [standard deviation (SD) of 0.003 rad/s], N2 (SD of 0.01 rad/s), N3 (SD of 0.05 rad/s), and N4 (SD of 0.1 rad/s).

In order to determine the alignment performance of the nonlinear least squares method for several alignment orientations from a fixed initial orientation, seven alignment orientations in terms of Euler angles were set as follows: A1 (1°/0°/−1° in the order of roll/pitch/yaw), A2 (45°/1°/−1°), A3 (−1°/45°/1°), A4 (1°/−1°/45°), A5 (45°/−45°/1°), A6 (1°/45°/−45°), and A7 (−45°/1°/45°). Regarding the fixed initial orientation for the MATLAB function *fsolve*, the quaternion ${\left[1,\;0,\;0,\;0\right]}^{T}$ was used for M1, and the identity matrix that corresponds to the aforementioned quaternion was used for M3 and M4.

For each of the tests, the results of the proposed method (M1) were compared with those of the other three methods (M2–M4). M2 is the DCM-based method introduced in [20] by using the pseudoinverse operation. In this paper, the pseudoinverse matrix is obtained using singular value decomposition (SVD). M3 is the same as M2 except that it uses the MATLAB function *fsolve* instead of the pseudoinverse operation. M4 is also the DCM-based method introduced in [21] that uses *fsolve*. To summarize, (i) M1 uses the quaternion while the other three use the DCM, (ii) M2 uses SVD while the other three use *fsolve*, and (iii) M4 is based on the gyroscopic angle, while the other three are based on angular velocity (Table 1).

### 3.3. Validation Using Experimental Data

**Figure 1:**
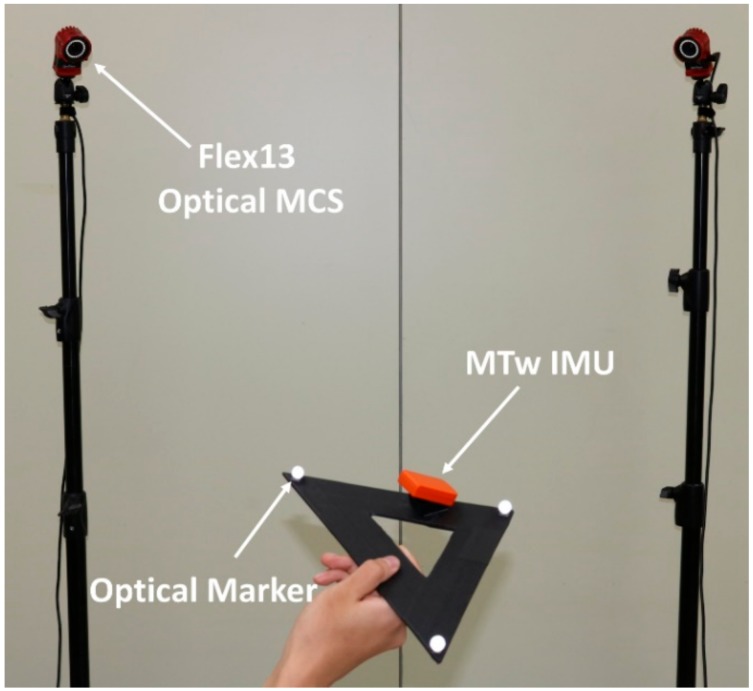
Experimental setup.

The purpose of the validation using experimental data in this chapter is to provide examples of actual alignment accuracy levels based on the specific IMU and MCS at our disposal. In addition to the aforementioned MTw IMU system, an OptiTrack Flex13 motion capture system (NaturalPoint, Inc., Corvallis, OR, USA) was used with the same sampling rate (i.e., 100 Hz), in order to obtain the angular velocities with respect to the MCS frame ωM (see Figure 1). For taking simultaneous measurements, the MTw sensor was mounted on top of a plastic right triangle ruler (with a 31 cm hypotenuse) and then three reflective markers from the Flex13 MCS were attached to each vertices of the ruler using double-sided adhesive tapes. These three markers form a plane that defines a unique orientation. Then, ωM’s were derived from the orientation.

Note that the MTw was mounted on the ruler with a specific alignment orientation which is the one that alignment methods estimate. The alignment orientation in terms of Euler angles was determined as 1.958°/−30.576°/44.347° in the order of roll/pitch/yaw. Since these angles were used as references for the calculation of estimation errors from the alignment method, they were determined using only very sophisticated data with particular careful attention, by applying the following conditions: (i) 3<||ω||<7 rad/s, (ii) −0.03<‖ωM‖−‖ωI‖<0.03 rad/s, and (iii) variation in distances between the markers should be less than 0.5 mm. From about 20 min of data recording (i.e., about 120,000 samples), only 13,564 samples that met the above conditions were input to the angular velocity-based methods (i.e., M1, M2, and M3) so that all three methods yielded almost same alignment estimation results. Accordingly, the averages of the results from M1, M2, and M3 were considered as the reference angles. The averaged variation and maximum variation from the reference angles were only 22 mdeg and 42 mdeg, respectively.

Two tests were conducted: TS (1.5<||ω||<2 rad/s, relatively slow) and TF (5<||ω||<6.5 rad/s, relatively fast). As in Section 3.2, the ruler which the MTw was mounted onto was moved by hand in a random manner. Ten trials were performed for each test, and each trial was set to 6000 samples of data after applying the same cutoff threshold as in Section 3.2.

## 4. Results

**Figure 2:**
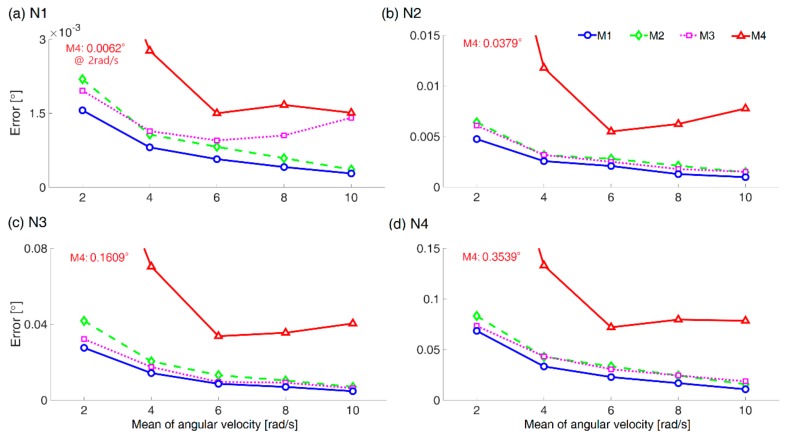
Averaged RMSE results from the simulated data.

With regard to the validation using the simulated data, Figure 2 shows the averaged root mean squared errors (RMSEs) from 70 trials (i.e., 10 trials × 7 alignment orientations) for each noise level from N1 to N4.

**Figure 3:**
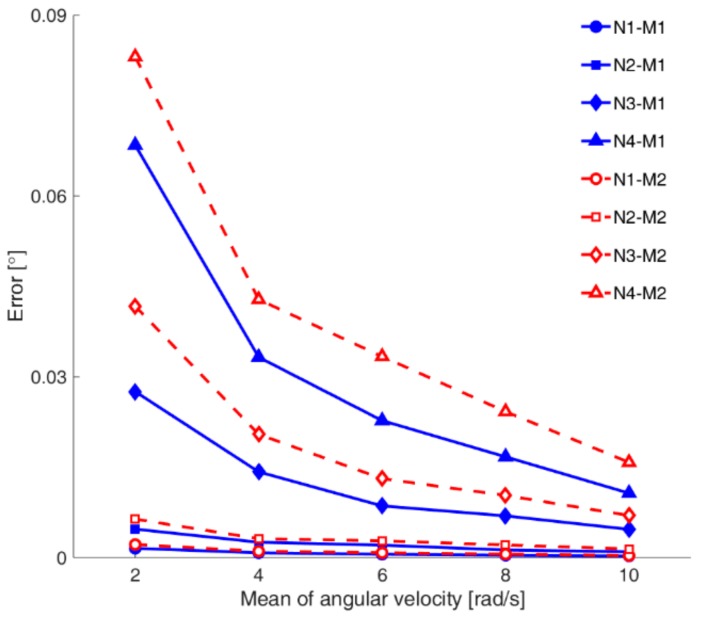
Comparison between estimation results from the simulated data, for M1 and M2.

Table 2 lists the detailed RMSEs from the simulated data in terms of the mean and standard deviation. It is true that in the noise level of the sensors for both IMU and MCS, there are large variations for each product. The average of 1400 RMSE data values for each method (i.e., 70 RMSE data × 5 different tests × 4 noise levels) is as follows: 11.46, 15.64, 14.31, and 57.02 mdeg for M1, M2, M3, and M4, respectively. Therefore, the order of accuracy by method is as follows: M1 > M3 ≈ M2 > M4. As shown in Figure 3, the proposed method M1 performed slightly better than M2 introduced in [20], consistently for all noise levels and angular velocities.

With regard to the validation using the experimental data, Table 3 shows the RMSEs in terms of the mean and standard deviation. Although the RMSEs were much higher than those from the simulated data due to addition of errors from the MCS measurement into the input data, M1 was slightly but consistently superior to M2 and M3. Furthermore, the standard deviations of M1 were smaller than those of M2 and M3.

## 5. Discussion and Conclusions

The performance of M4 was lower than those of the other three methods. This result is in good agreement with the finding in [22]. The inferior performance of M4 is possibly associated with the use of gyroscopic angles rather than angular velocities. Note that M4 is the only method that uses the angle information for estimation. The strapdown integration process to obtain the gyroscopic angles is inherently prone to drift errors. Then, the frame transformation based on erroneous angles may result in errors in alignment estimation. Although [21] argued that the gyroscopic angle provides a better S2N ratio than the angular velocity, the performance of M4 was lower than those of the other three methods for all noise levels (i.e., N1 to N4).

If we consider that the only difference between M1 and M3 is the orientation representation (see Table 1), it may be said that the quaternion chosen by M1 is better than the DCM chosen by M3 for the estimation of alignment orientation. For the quaternion and DCM, the numbers of component (to be estimated) are four and nine, while the numbers of constraints are one and six, respectively. The fewer number of components and constraints of the quaternion may yield better estimates within the same data sets.

As the angular velocity increased, the error tended to decrease, in general, in both the simulated data (see Table 2 and Figure 2) and the experimental data (see Table 3). This may be because the influence of the noise is relatively reduced when the angular velocity increases at the same noise level. In the case of M4, this tendency was irregular. This irregularity is also suspected to be related to the strapdown integration process to obtain angles for M4.

As the noise level increased, the error tended to increase, which is simply natural (see Table 2). A noticeable factor is that the order of accuracy by method was the same for all noise levels. This means that one method cannot be said to be more sensitive than the other depending on the S2N ratio. Specifically, with regard to *C1* on the S2N ratio issue, our result does not support the description in [21], that is, the gyroscopic angle compared to the angular velocity provides a good S2N ratio. 

With regard to *C2*, the applied solvers were not critical in general. However, we could find some effects related to the use of the nonlinear least square method *fsolve* in M3. See Table 4 for the coefficients of variance (CVs) between the RMSE results based on the simulated data, from seven different alignment orientations, i.e., A1 to A7, where each RMSE result used for the CV calculation is the average of the RMSEs from 10 trials. The CVs were as small as 0.5 or less in all methods, except 0.64 of T4 and 0.62 of T5 from M3 in N1. Small CV means that the difference of estimation performance by alignment orientation is small. Averaged RMSEs from 10 trials from A1 to A7, in case of T4 from M3 in N1 were 0.43, 0.52, 0.64, 0.50, 1.76, 1.94, and 1.57 mdeg, respectively; and those in case of T5 from M3 in N1 were 0.34, 0.94, 0.90, 0.88, 2.57, 2.54, and 1.73 mdeg, respectively. Note that, in both cases, the RMSEs were high for A5, A6, and A7 when the alignment orientations were quite different from the initial orientation through two 45° rotations. Along with the CV distinction, as shown in Figure 2, the RMSE from M3 in N1 increased as the angular velocity increased from T4 to T5 compared to the RMSE at T3. Considering that the difference between M2 and M3 exists only in the solver (see Table 1), obviously the estimation difference comes from the difference of the solver, which is consistent with the description in [22], although [22] compares M2 and M4. In the case of M1, low CV was maintained in all cases. Even though both M1 and M3 use the *fsolve*, M3 based on DCM was sometimes affected by alignment orientation but M1 based on quaternion was not affected. This also implies the superiority of quaternion to DCM for orientation alignment problem.

In this study, we proposed a quaternion-based local frame alignment method where a quaternion is used to represent the orientation. The performance of the proposed method was compared with those of three other methods. Within our limited test data, the proposed method performed the best among the four methods. We expect that the fewer number of components and constraints of the quaternion in the proposed method compared to number of components and constraints of the DCM may result in better accuracy. Owing to its high accuracy and easy setup, the proposed method can be effectively used for local frame alignment between an IMU and an MCS.

## Figures and Tables

**Table 1:** Summary of methods.

Method	Representation	Solver	Concept	Reference
M1	Quaternion	*fsolve*	Angular velocity transformation	Proposed
M2	DCM	Pseudoinverse	Angular velocity transformation	[20]
M3	DCM	*fsolve*	Angular velocity transformation	Modified from [20]
M4	DCM	*fsolve*	Angle transformation	[21]

**Table 2:** RMSE results from the simulated data (unit: mdeg): mean (standard deviation) from M1 (quaternion-based proposed method), M2 (method by pseudoinverse), M3 (method modified from M2), and M4 (method using angle transformation).

**N1**					**N2**				
	**M1**	**M2**	**M3**	**M4**		**M1**	**M2**	**M3**	**M4**
T1	**1.56** (0.62)	2.19 (0.90)	1.96 (0.83)	6.23 (4.18)	T1	**4.74** (2.83)	6.40 (3.58)	6.08 (3.07)	37.87 (33.07)
T2	**0.81** (0.50)	1.07 (0.50)	1.14 (0.59)	2.77 (1.81)	T2	**2.56** (0.91)	3.15 (1.59)	3.17 (1.56)	11.78 (8.54)
T3	**0.57** (0.41)	0.82 (0.41)	0.95 (0.51)	1.50 (0.78)	T3	**2.08** (0.80)	2.80 (0.99)	2.50 (1.22)	5.49 (2.59)
T4	**0.41** (0.26)	0.59 (0.32)	1.05 (0.77)	1.67 (0.82)	T4	**1.28** (0.73)	2.11 (0.91)	1.78 (0.99)	6.23 (2.49)
T5	**0.28** (0.13)	0.36 (0.19)	1.41 (0.93)	1.51 (0.94)	T5	**0.98** (0.47)	1.46 (0.95)	1.51 (0.82)	7.76 (7.43)
**N3**					**N4**				
	**M1**	**M2**	**M3**	**M4**		**M1**	**M2**	**M3**	**M4**
T1	**27.55** (11.95)	41.72 (20.04)	32.15 (13.72)	160.90 (98.42)	T1	**68.43** (30.59)	83.07 (39.40)	73.26 (36.50)	353.92 (250.67)
T2	**14.24** (5.89)	20.46 (8.69)	17.46 (7.63)	70.37 (50.40)	T2	**33.26** (11.55)	42.83 (20.07)	43.23 (19.72)	133.06 (61.43)
T3	**8.59** (4.39)	13.11 (5.90)	9.58 (4.61)	33.68 (18.82)	T3	**22.74** (9.28)	33.37 (14.63)	30.54 (13.37)	71.93 (32.84)
T4	**6.94** (3.28)	10.33 (3.70)	9.18 (5.05)	35.50 (21.17)	T4	**16.73** (8.08)	24.23 (13.40)	24.37 (15.57)	79.54 (34.21)
T5	**4.70** (2.94)	7.00 (3.65)	6.30 (3.50)	40.38 (38.54)	T5	**10.70** (5.50)	15.81 (8.33)	18.53 (10.90)	78.38 (48.74)

**Table 3:** RMSE results from the experimental data (unit: mdeg): mean (standard deviation) from M1, M2, M3, and M4.

	M1	M2	M3	M4
TS (relatively slow)	215 (80)	287 (103)	343 (159)	440 (132)
TF (relatively fast)	168 (58)	177 (63)	197 (63)	616 (364)

**Table 4:** Coefficients of variation between RMSE results based on the simulated data, from seven different starting points.

**N1**					**N2**				
	**M1**	**M2**	**M3**	**M4**		**M1**	**M2**	**M3**	**M4**
T1	0.14	0.08	0.11	0.44	T1	0.16	0.20	0.06	0.29
T2	0.25	0.12	0.19	0.22	T2	0.14	0.15	0.09	0.29
T3	0.28	0.27	0.24	0.11	T3	0.10	0.16	0.20	0.20
T4	0.22	0.19	0.64	0.13	T4	0.17	0.22	0.21	0.20
T5	0.10	0.15	0.62	0.18	T5	0.13	0.16	0.17	0.24
**N3**					**N4**				
	**M1**	**M2**	**M3**	**M4**		**M1**	**M2**	**M3**	**M4**
T1	0.15	0.13	0.17	0.21	T1	0.15	0.12	0.25	0.12
T2	0.13	0.11	0.12	0.24	T2	0.13	0.17	0.13	0.14
T3	0.18	0.10	0.15	0.25	T3	0.16	0.22	0.13	0.13
T4	0.19	0.19	0.20	0.29	T4	0.14	0.18	0.20	0.16
T5	0.26	0.25	0.12	0.14	T5	0.18	0.23	0.27	0.15

## Supplementary Materials

Supplementary source code and example data associated with this article can be found at http://www.mdpi.com/1424-8220/18/11/4003/s1.
